# Manufacture of Gold Foil in St. Louis, Mo.

**Published:** 1848-01

**Authors:** 


### MANUFACTORY OF GOLD FOIL IN ST. LOUIS, MO.

We feel pleasure in drawing the attention of the profession in this section of the country, to the card of Mr. James S. Pool, manufacturer of Dentist's Gold Foil. We freely add our testimony, in behalf of the excellent article of Foil from his manufactory, having used it during the last year. As Mr. Pool is the only manufacturer here, and the first to locate among us, we bespeak for him a support from the profession.—St. L. Ed.

